# A cone-beam computed tomography based workflow for online adaptive ultra-hypofractionated radiotherapy of prostate cancer

**DOI:** 10.1016/j.phro.2025.100869

**Published:** 2025-11-17

**Authors:** Miriam Eckl, Nour Alfakhori, Marvin Willam, Hans Oppitz, Constantin Dreher, Michael Ehmann, Judit Boda-Heggemann, Frank A. Giordano, Jens Fleckenstein

**Affiliations:** a Department of Radiation Oncology, University Medical Centre Mannheim, Heidelberg University, Germany; b DKFZ-Hector Cancer Institute, University Medical Centre Mannheim, Mannheim, Germany; c Mannheim Institute for Intelligent Systems in Medicine (MIISM), Medical Faculty Mannheim, Heidelberg University, Mannheim, Germany; d Junior Research Group “Intelligent Imaging for adaptive Radiotherapy”, Mannheim Institute for Intelligent Systems in Medicine (MIISM), Heidelberg University, Germany

**Keywords:** Prostate cancer, Adaptive radiation therapy, Ultra-hypofractionation, SBRT, Hypersight CBCT

## Abstract

**Background and purpose:**

Cone beam computed tomography (CBCT)-based approaches for online adaptive radiation therapy (oART) have recently become clinically available for ultra-hypofractionated prostate stereotactic body radiation therapy (SBRT). This work assessed the changes of relevant dose-volume-histogram (DVH) parameters and their robustness against morphologic variations during adaptation.

**Materials and methods:**

Sixteen prostate cancer patients were treated with oART in an SBRT regimen (40 Gy in 5 treatment sessions (Tx) to the clinical target volume (CTV), PACE-B constraints). Two CBCTs were acquired daily: CBCT1 for adaptive planning and CBCT2 after adaptation for position verification. Adapted plans optimized on CBCT1 (ART_CBCT1_) were recalculated on CBCT2 (ART_CBCT2_) and compared to treatment plans on CBCT1 after image guidance (IGRT_CBCT1_) for relevant DVH metrics: V_40Gy_(CTV), V_37Gy_(bladder), V_36Gy_(rectum). Spearman’s rank coefficients r with p-values (5% significance level) were determined to analyze correlations between adaptation time (ΔT) and bladder filling as well as Tx and median prostate volume.

**Results:**

oART improved median V_40Gy_(CTV) from 86% in IGRT_CBCT1_ to 94% in ART_CBCT2_. Inter-fractional prostate swelling ($r_{Tx,Vol(prostate)}=0.98,p=0.005)$ was responsible for CTV deviations. Bladder filling ($r_{\Delta T,Vol(bladder)}=0.34,p=0.002$) and rectal gas migration during the median adaptation time ΔT=24.0min increased V_37Gy_(bladder) from 4.9 cm^3^ in ART_CBCT1_ to 6.5 cm^3^ in ART_CBCT2_ and V_36Gy_(rectum) from 0.5 cm^3^ to 0.6 cm^3^ and led to 10 constraint violations, each.

**Conclusion:**

Compared to IGRT, daily oART substantially improved CTV coverage. Besides inter-fractional prostate swelling, constraint violations originated from seminal vesicles motion, rectal gas or bladder filling during adaptation. Treatment adaptation times should therefore be minimized whenever possible.

## Highlights

• Adaptation improved quality of ultra-hypofractionated prostate cancer radiotherapy.
• A prostate volume swelling of median 18% in treatment session five was observed.
• Median adaptation time was 24 min.
• 82% of non-adaptive plans did not fulfill the minimum target volume coverage.
• In 10 of 80 sessions bladder filling during adaptation lead to constraint violations.

## Graphical abstract

**Figure:**
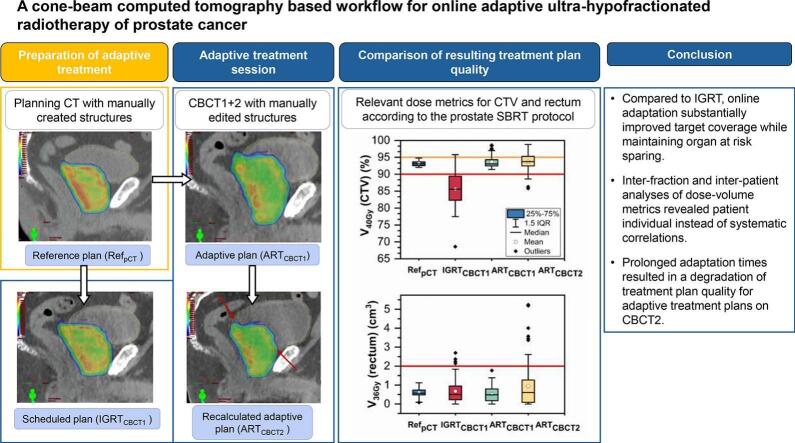

## 1 Introduction

Over the past five years, low or intermediate-risk prostate cancer patients have successfully been treated with ultra-hypofractionated radiation therapy (uHF) protocols, such as the PACE, HYPO-RT or Hypo-FLAME trial [[1], [2], [3]]. These treatments are characterized by fraction doses over 5 Gy and yield toxicities comparable to normal or moderately hypofractionated (mHF) treatment protocols alongside promising relapse-free survival rates [[4], [5], [6], [7]]. While image-guided radiation therapy (IGRT) has proven to substantially compensate for inter-fractional organ translations and rotations [8], it cannot account for prostate and seminal vesicles (SV) deformations or variations in organ at risk (OAR) filling [9,10]. For mHF prostate cancer treatments with planning target volume (PTV) margins of approximately 10 mm, IGRT or offline adaptive radiation therapy (ART) approaches were mainly applied [[11], [12], [13]]. Treatment approaches included plan libraries [14], adapt-to-position approaches [15] or patient-specific hybrid techniques [16,17]. However, if smaller margins [18] or uHF protocols are pursued, online ART (oART) is recommended to maintain target coverage and avoid overdoses to OARs [19,20].

Initial technical obstacles to a widespread introduction of oART like insufficient daily imaging quality and a demand for treatment automation have recently been overcome [[21], [22], [23]]. uHF oART for prostate cancer has predominately been performed at magnetic resonance imaging (MRI)-based linear accelerators (linacs) [[24], [25], [26], [27]]. The first system with a fully integrated workflow for cone beam computed tomography (CBCT)-imaging based oART emerged in 2019 [28] followed by the clinical introduction of high quality CBCT-imaging in 2023 [29].  Although MRI-guided and robotic radiosurgery treatments [30] offer the advantages of superior soft-tissue contrast or intra-fractional gating, CBCT-guided oART is characterized by faster imaging times [28,31,32]. Furthermore, the recently established high quality CBCT imaging devices [29] removed the need for creating synthetic CT for electron density mapping [33].

This study analyzed a prostate patient cohort that received an uHF, fully oART with a high-quality CBCT used directly for treatment plan adaptation. The aim of this work was to provide a realistic estimate of the resulting dose distributions based on the adapted plan and a CBCT acquired directly prior to dose delivery. Furthermore, potential origins for deviations of relevant dose-volume-histogram metrics within the adaptation process such as the treatment session, adaptation time and patient- or morphology-specific properties were examined.

## 2 Materials and methods

### 2.1 Patient cohort

Sixteen patients with primary prostate cancer of low or intermediate risk, who received treatment between July 2024 and January 2025, were evaluated retrospectively after institutional review board approval (reference number 2023–557). Patient characteristics are displayed in Tab.1.

**Table 1:** Patient characteristics with number of patients n and median values (quartile one Q1, quartile three Q3). The total cohort comprised n = 16 patients.

		n	median (Q1, Q3)
Age (years)			71 (67, 75)
Pre-treatment PSA (ng/ml)			6.9 (5.2, 8.5)
Gleason Score	6 (3 + 3)	1	
	7 (3 + 4)	15	
Tumor stage	T1c	12	
	T2a-T2c	4	
Volume (cm^3^)	prostate		45.7 (40.5, 59.4)
	SV		14.4 (12.5, 20.1)
Body-mass-index BMI			26.1 (25.6, 27.2)
Comorbidities	Diabetes	1	
	Cardiovascular	6	
	Hypertension	5	

All patients were treated with uHF oART according to PACE-B regimen (36.25/40 Gy to PTV and clinical target volume (CTV) in five fractions) [1] at an Ethos therapy system (v2.0, Varian Medical Systems, Palo Alto, USA). Treatment plans were normalized to $V_{40Gy}\left(CTV\right)=92-93\%$, based on the remaining OAR constraints. All patients received multiparametric MRI for lesion detection and urethra localization. Locoregionally registered transversal and sagittal T2-weighted turbo spin-echo sequences were used for prostate, SV and urethra segmentation.

Thirty minutes prior to treatment planning CT (pCT) and all treatment sessions (Tx), patients were requested to empty their rectum with an enema, empty their bladder and subsequently drink 500 ml water. Furthermore, all patients were repeatedly instructed to avoid flatulent food and thoroughly comply with the preparation guidelines.

### 2.2 Online adaptive treatments

pCT were acquired with a standard CT-scanner (Brilliance Big Bore, Philips Healthcare, Amsterdam, Netherlands) 7.7 ± 2.5 days prior to the first Tx. Initial automated contouring and, if necessary, manual corrections on pCT as well as treatment planning were performed in the treatment planning system (ETM, Ethos treatment management, v3.0). The prostate plus a proximal 1 cm intersection with the SV were expanded by 2 mm in order to create the CTV. The PTV was generated by adding an additional isotropic 3 mm margin to the CTV, except for the posterior direction with 2 mm.

The five adaptive treatment fractions were delivered daily but always contained a weekend within the treatment course. Patients were imaged with a novel CBCT imaging device (HyperSight, Varian Medical Systems) using the standard pelvis preset (125 kV, 470 mAs, field-of-view diameter 53.8 cm) and the provided iterative Acuros reconstruction mode. Hypersight CBCT possessed a resolution of $1.05\times 1.05\;{cm}^{2}$, a slice thickness of 2 mm and CBCT numbers agreed within less than ±35HU with pCT information [34].

All patients received two CBCT per Tx. On CBCT1, five influencer structures (prostate, SV, bladder, rectum, bowel) were automatically segmented and subsequently manually adjusted by medical doctors (MD). In total, six MDs were involved in the adaptation process based upon clinical availability. As a measure for inter-observer variability of target volume segmentation among all MDs the ratio k of the CTV-volume of a Tx=s(s=2,3,4) and the mean of the CTV-volumes of the previous (s-1) and the subsequent Tx (s+1)

(1) $$k=\frac{2\times {V(CTV)}_{Tx=s}}{{V(CTV)}_{Tx=s-1}+\;{V(CTV)}_{Tx=s+1}}$$

were assessed.

Subsequently, margins for CTV and PTV were created and other OARs, such as urethra, were manually segmented with a co-registered pCT and/or MRI. The adaptive plan was optimized on CBCT1 with the same planning directives as the reference treatment plan Ref_pCT_ and dose was calculated with the same CBCT-number-to-electron density calibration. Subsequent to successful online QA (Mobius 3D, v4.0.2) and plan acceptance, CBCT2 was acquired for a translational re-positioning plan delivery. Dose was delivered with 12-field (n=12) or 9-field (n=4) IMRT.

### 2.3 Retrospective CBCT-based dose analysis

Based on the resulting shift-vector between the two CBCT per Tx, all relevant OAR and the target structures were transferred from CBCT1 to CBCT2 and manually corrected, whenever needed (Velocity, v4.1, Varian Medical Systems). The dose distribution of the reference plan Ref_pCT_ was recalculated (Eclipse v17.0, Varian Medical Systems) with its original treatment iscocenter (ISO). The IGRT scenario (IGRT_CBCT1_) was acquired by determining the dose distribution of the reference plan with the applied ISO-shift on CBCT1 (IGRT_CBCT1_). The adaptive treatment plan on CBCT1 (ART_CBCT1_) was recalculated accordingly. Lastly, based on the shift vector between CBCT1 and CBCT2, the dose distribution of the adapted treatment sequence was determined on CBCT2 (ART_CBCT2_). This led to 256 dose distributions: 16 patients with five Tx and three CBCT–treatment-plan combinations (IGRT_CBCT1_, ART_CBCT1_, ART_CBCT2_) plus sixteen Ref_pCT_.

The following dose-volume-histogram (DVH) metrics of the uHF protocol were extracted: $V_{40Gy}\left(CTV\right)$, $V_{36.25Gy}\left(PTV\right)$, $V_{37Gy}\left(bladder\right)$ and $V_{36Gy}\left(rectum\right)$. DVH metrics were compared to mandatory PACE-B dose volume constraints: $V_{40Gy}\left(CTV\right)>90\%$, $V_{36.25Gy}\left(PTV\right)>90\%$, $V_{37Gy}\left(bladder\right)<10\;{cm}^{3}$ and $V_{36Gy}\left(rectum\right)<2\;{cm}^{3}$. Organ volumes and Dice similarity coefficients (DSC) of the prostate and SV between CBCT1 and CBCT2 were extracted. Median values (quartile one Q1, quartile three Q3) were reported for all quantities.

To analyze potential inter-factional dependencies, Spearman’s rank correlation coefficients (r) along with tests for significance on a 5% level (p) between the Tx (Tx1-Tx5) and the following (per session) metrics were determined: Median adaptation time ΔT (between the acquisition of CBCT1 and CBCT2) as well as median volumes of bladder, rectum and prostate on CBCT1. Moreover, the correlation between ΔT and bladder filling ΔVol(bladder) during adaptation for all Tx as well as potential changes in relevant DVH metrics were evaluated. Lastly, in order to identify patient-specific origins for dose deviations during adaptation, three representative sessions were analyzed that reflected the largest reduction of CTV coverage, the highest rectum dose increase and the largest bladder filling during adaptation.

## 3 Results

**Fig. 1:**
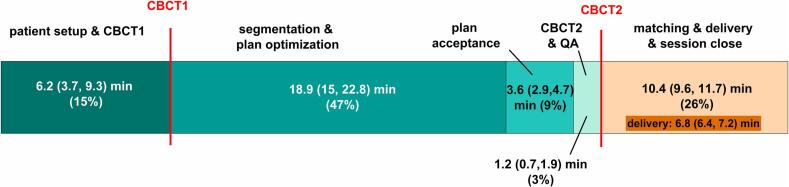
Workflow steps and their median duration (Q1, Q3) during online adaptive treatment sessions.

Documented times of all relevant workflow steps within the Tx are displayed in Fig. 1.

The overall adaptation time was $\Delta T=24.0\;\left(20.3,28.6\right)\;min$. The fastest MD required $\Delta T=19.3\;\left(17.6,23.4\right)\;min$ (n=20 sessions) and slowest needed $\Delta T=32.7\;\left(32.7,32.8\right)\;min$ (n=2 sessions). Inter-observer target segmentation variability was k=101%(96%,106%). The adaptive plan (ART_CBCT1_) instead of the scheduled plan (IGRT_CBCT1_) was always selected for treatment. The monitor units (MU) of the 16 Ref_pCT_ were 3441.4±428.4MU, while all 80 adaptive plans had 3434.8±390.3MU. Shift-vectors between CBCT1 and CBCT2 were $0.1\;\left(-0.8,0.8\right)mm$ in left–right, $-1.2\left(-2.2,-0.8\right)mm$ in inferior-superior and $0.3\;\left(-0.2,1.2\right)mm$ in cranio-caudal direction.

**Fig. 2:**
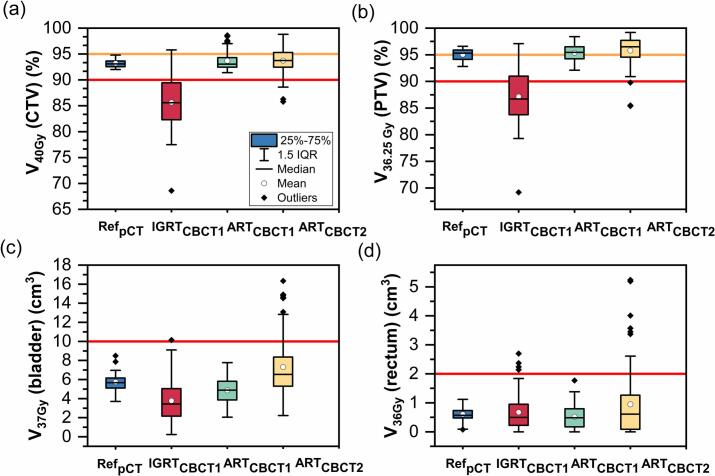
Boxplots for the four most relevant DVH metrics V_40Gy_(CTV) (a), V_36.25Gy_(PTV) (b), V_37Gy_(bladder) (c) and V_36Gy_(rectum) (d). Each metric was grouped by the four different treatment plan types of the reference plan Ref_pCT_ (n = 16), the scheduled plan IGRT_CBCT1_ (n = 80), the adapted plan on both CBCT ART_CBCT1_ (n = 80) and ART_CBCT2_ (n = 80). The red and orange lines indicate mandatory and optimal thresholds for fulfilling the DVH metric according to PACE-B protocol. (For interpretation of the references to colour in this figure legend, the reader is referred to the web version of this article.)

In Fig. 2 boxplots of the four relevant DVH metrics are displayed for the four treatment plan types. Compared to IGRT_CBCT1_, ART_CBCT1_ improved median $V_{36.25Gy}\left(PTV\right)$ from 87% to 96% (panel (b)). Due to its normalization V_40Gy_(CTV) always fulfilled its protocol tolerances for Ref_pCT_ and ART_CBCT1_. The reported values in panel (a) were higher than the initial normalization due to the recalculation in Eclipse. The deviation of D_50%_(CTV) between Eclipse and ETM was 0.2%(0.1%,0.2%). While panels (a) and (b) indicate a relevant improvement of target volume DVH metrics through oART, a degradation of DVH parameters between ART_CBCT1_ and ART_CBCT2_ can be seen in all panels. The number of constraint violations for all treatment scenarios is displayed in Tab.2.

**Table 2:** Number of treatment sessions (Tx) in which a given constraint was violated (out of 16 initial treatment plans and for 80 Tx). In brackets are the constraint violations by Tx from Tx1 to Tx5.

DVH constraint	Ref_pCT_	IGRT_CBCT1_	ART_CBCT1_	ART_CBCT2_
V_40Gy_(CTV) ≥ 90%	0	65 (6,13,16,15,15)	0	7 (0,1,2,2,2)
V_36.25Gy_(PTV) ≥ 90%	0	54 (3,11,14,13,13)	0	3 (1,0,0,0,2)
V_37Gy_(bladder) < 10 cm^3^	0	1 (1,0,0,0,0)	0	10 (1,2,3,2,2)
V_36Gy_(rectum) < 2 cm^3^	0	4 (2,0,0,1,1)	0	10 (1,1,4,3,1)

While ART_CBCT1_ resulted in no constraint violation for any DVH metric, 10 constraint violations of up to $V_{37Gy\left(bladder\right)}=16.2\;{cm}^{3}$ and $V_{36Gy(rectum)}=5.3{\;cm}^{3}$ were found in ART_CBCT2_.

In Tab.3 ΔT as a function of Tx is displayed. No significant correlation (see Tab. S1 for correlation coefficients r and p-values) between Tx and ΔT was observed. The prostate volume revealed a highly correlated and significant increase with treatment progress of up to $18\%\;\left(7\%,24\%\right)$ in Tx5. The inter-modality difference in prostate segmentation on CBCT1 of Tx1 and the corresponding pCT was 2%(-2%,4%) while the daily prostate volume difference between CBCT2 and CBCT1 of Tx1 was 0%(0%,1%). For IGRT_CBCT1_, this volume increase led to a reduced CTV coverage: V_40Gy_(CTV) for all Tx1 was 91% (89%, 92%) while it was 83% (88%, 81%) for all Tx5. For ART_CBCT1_ this decrease in dose coverage was no longer present with $V_{40Gy}\left(CTV\right)=93\%\;\left(92\%,\;94\%\right)$ for Tx1 and 93% (92%, 95%) for Tx5. Both prostate and SV showed intra-fractional motion during ΔT but no dependency of DSC and Tx was observed.

**Table 3:** Median values (Q1, Q3) of relevant parameters for the individual treatment sessions (Tx): The adaptation time ΔT, the relative prostate volume change ΔVol(prostate) from CBCT1 of Tx1, the relative bladder volume change ΔVol(bladder) from pCT, the relative rectum volume change ΔVol(rectum) from pCT and the Dice similarity coefficients (DSC) between CBCT1 and CBCT2 of prostate and SV.

treatment session	Tx1	Tx2	Tx3	Tx4	Tx5
adaptation time ΔT (min)	24.1 (21.8, 26.2)	23.9 (21.9, 27.6)	24.3 (18.1, 31.4)	24.1 (22.0, 29.6)	24.4 (19.0, 28.9)
ΔVol(prostate) from CBCT1 (%)	0 (0, 0)	13 (1, 19)	13 (9, 22)	12 (7, 21)	18 (7, 24)
ΔVol(bladder) from pCT (%)	60 (8, 82)	4 (−20, 73)	35 (10, 97)	26 (−35, 63)	−17 (−34, 72)
ΔVol(rectum) from pCT (%)	1 (−11, 31)	−2 (−11, 4)	3.6 (−9, 27)	3 (−9, 23)	8 (−3, 19)
DSC_CBCT1,CBCT2_(prostate)	0.97 (0.96, 0.97)	0.96 (0.94, 0.97)	0.96 (0.95, 0.96)	0.96 (0.92, 0.97)	0.96 (0.92, 0.98)
DSC_CBCT1,CBCT2_(SV)	0.86 (0.83, 0.89)	0.90 (0.82, 0.91)	0.87 (0.82, 0.89)	0.85 (0.83, 0.87)	0.88 (0.80, 0.90)

The patient with the largest prostate motion possessed a ${DSC}_{CBCT1,CBCT2}\left(prostate\right)=0.89\;\left(0.89,0.92\right)$ and the patient with least motion had a ${DSC}_{CBCT1,CBCT2}\left(prostate\right)=0.98\;\left(0.98,0.99\right)$. The patient with the largest or least SV deviations resulted in a ${DSC}_{CBCT1,CBCT2}\left(SV\right)=0.77\;\left(0.62,0.84\right)$ or ${DSC}_{CBCT1,CBCT2}\left(SV\right)=0.91\;\left(0.89,0.95\right)$, respectively. A weak correlation between CTV-volume and DSC of the two CBCT was observed. However, the CTV-volume was not correlated to a change in target coverage. Bladder and rectum volume differences between CBCT2 of the respective Tx and the pCT volume revealed no relevant correlation with Tx.

The change in rectum-volume during adaptation was $1.8\left(-0.7,3.8\right)\;{cm}^{3}$. Among all Tx, a relevant gas appearance was detected in 21 (CBCT1) or 30 (CBCT2) Tx. In five of those 30 Tx a pronounced increase of rectum gas between CBCT1 and CBCT2 occurred that caused $V_{36Gy}\left(rectum\right)$ to increase by more than 2.5 cm^3^. A relevant gas increase was not concentrated on individual patients but occurred randomly (P2Tx4, P4Tx2, P7Tx1, P9Tx3, P15Tx5).

ΔT was correlated weakly but statistically significant with bladder filling $\Delta Vol\left(bladder\right)$ during adaptation. However, no statistically significant correlation was found between ΔT and any change in DVH metrics during adaptation.

**Fig. 3:**
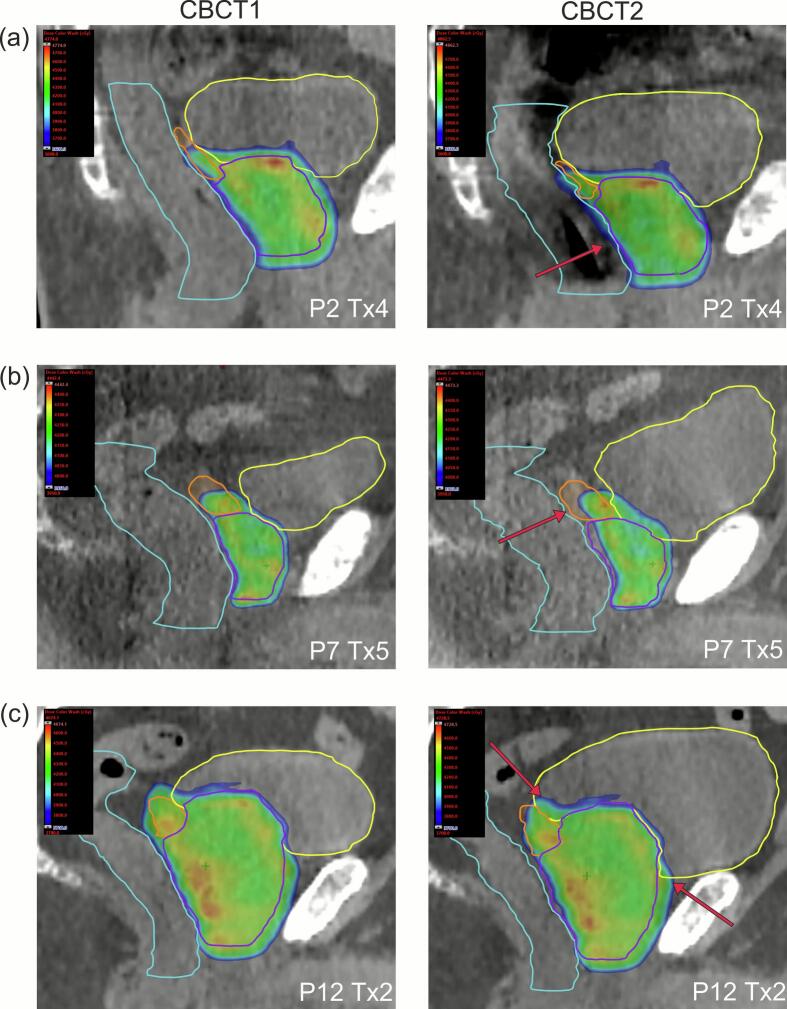
Sagittal CT planes containing dose distributions of ART_CBCT1_ (left column) and ART_CBCT2_ (right column) for three selected patients with the structures bladder (yellow), rectum (turquoise), SV (orange) and prostate (purple). Minimum isodose was set to 36 Gy, 40 Gy and 37 Gy to illustrate each constraint violation in the respective treatment session (Tx) on CBCT2 of V_36Gy_(rectum) for P2 (a), V_40Gy_(CTV) for P7 (b) and V_37Gy_(bladder) for P12 (c). Red arrows represent relevant morphological changes in high dose regions. (For interpretation of the references to colour in this figure legend, the reader is referred to the web version of this article.)

In Fig. 3 three representative treatment scenarios are presented:

Patient P2 showed the highest median $V_{36Gy}\left(rectum\right)=1.7\;{cm}^{3}$ on CBCT2. The largest constraint violation occurred after adaptation in Tx4 with $V_{36Gy}\left(rectum\right)=4.0\;{cm}^{3}$ due to rectum gas resulting in an anterior displacement of the rectal wall into the high-dose volume (panel (a)).

Patient P7 had the worst $V_{40Gy}\left(CTV\right)=90\%\left(90\%,92\%\right)$ coverage after adaptation on CBCT2 and the cohort‘s smallest prostate volume (31.2 cm^3^). Moreover, this patient was characterized by a relevant bladder filling ${\Delta Vol}_{CBCT1,CBCT2}\left(bladder\right)=122\;\left(67,130\right){cm}^{3}$ and a low ${DSC}_{CBCT1,CBCT2}\left(SV\right)=0.77\;\left(0.57,0.88\right)$. This substantial movement of SV and bladder filling was responsible for the reduced CTV coverage in Tx5 (panel (b)).

Patient P12 (panel (c)) was treated with the longest adaptation time $\Delta T=33\left(32,41\right)\;min$ which resulted in a bladder filling of $122\left(99,239\right)\;{cm}^{3}$. Nevertheless, all DVH metrics were fulfilled on all CBCT2, except for Tx2 with $V_{37Gy}\left(bladder\right)=14.5\;{cm}^{3}$ which resulted from a large filling of the bladder during adaptation ($163\;{cm}^{3}$ on CBCT1, $393\;{cm}^{3}$ on CBCT2).

## 4 Discussion

This study aimed to analyze a patient cohort treated with a CBCT-based adaptive uHF protocol, evaluating dose pre- and post-adaptation on CBCT. ART_CBCT1_ treatment plans fulfilled all mandatory clinical dose-volume constraints, thus proving the capability of oART to reproduce the initially accepted dose distribution of the Ref_pCT_. Although IGRT_CBCT1_ was able to achieve adequate OAR sparing for $V_{37Gy}\left(bladder\right)$ and $V_{36Gy}\left(rectum\right)$ constraints, CTV coverage was insufficient in 54 of 80 treatment sessions.

CTV constraint violations occurred less for Tx1 than for Tx2-Tx5, indicating that the prostate swelling from Tx2-Tx5 was the main reason for this behavior. Since this swelling has not been reported for normo-fractionated prostate RT we postulate that it is a prostate–SBRT-specific issue. Prostate oART may therefore be less beneficial in normo-fractionated-RT.

Locoregional edema or radiation-induced inflammation could potentially induce prostate swelling [35,36]. No patient in this study received androgen deprivation theory (ADT) during RT. The volume increase was, among organ deformation, one of the reasons why the IGRT_CBCT1_ scenario led to lower target coverage than ART_CBCT1_. Studies of prostate SRBT at MRI linacs demonstrated prostate mean/median volume changes between 12–15% [[35], [36], [37]] with a similar convergence towards Tx5. The initial offset of the median CBCT volume versus pCT indicated a systematic deviation between the two imaging modalities.

Ratnakumeran et al. [38] reported that any genitourinary (GU) and gastrointestinal (GI) CTCAE grade 2+ toxicity could occur at $D_{max}\left(bladder\right)>40\;Gy$ or $D_{max}\left(rectum\right)>38\;Gy$. Therefore, relevant contributions $V_{37Gy}\left(bladder\right)>10\;{cm}^{3}$ or $V_{36Gy}\left(rectum\right)>2\;{cm}^{3}$ could result in grade 2 + toxicity. Target doses on the other hand were high in PACE-B (for $\frac{\alpha }{\beta }=2$: EQD2=84Gy, BED=168Gy) and biochemical clinical failure event–free rate after 5-years was 96% [39]. Therefore, a minor target violation was considered less problematic as long as no extended under-dosed volumes were present.

Available literature on adaptive prostate SBRT [40,41] particularly for CBCT-based oART [42], confirmed dose-related benefits of oART over IGRT. However, the results of ART_CBCT2_ implied that the adaptation process itself led to a partial loss of treatment quality due to morphological changes such as bladder filling, rectal gas dynamic and altered SV position. Shifts of the prostate, as frequently reported in any direction, could additionally originate from muscle relaxation [43] but could be corrected through IGRT.

Brennan et al. demonstrated an increased number of protocol violations with time by means of pre- and post-treatment MRI scans, causing DVH metrics to exceed their maximum mainly for bladder, rectum and urethra in 24–45% of all Tx [24]. A similar trend of treatment plan quality deterioration from ART_CBCT1_ to ART_CBCT2_ was described by Fischer et al. [44] for a normofractionated protocol with ΔT=25min. Mannerberg et al. [45] identified 2–4% coverage loss for PTV margins of 3/5 mm (posterior/else) with a ΔT=30min between two MRIs in an uHF prostate RT. The only other research study comprising an CBCT-based workflow identified benefits over IGRT mainly for PTV [40], with for instance 9% for $V_{36.25Gy}\left(PTV\right)$ which is in close agreement to the findings in this work.

The ΔT=24min was longer than reported times of CBCT-based oART for mHF prostate protocols with ΔT=11.9min [46] which mainly originated from more complex segmentation and subsequent higher degree of plan optimization. Corresponding ΔT at MRI linacs were reported to be up to 45 min [26] or 34–86 min [47]. No “familiarization” effect of involved personnel that would result in a faster adaptation time with progressing Tx was observed in this study.

The evaluation of inter-fraction data for the bladder volume revealed no correlation, implying that during only five fractions patients acquired no specific learning behavior regarding their bladder filling. Although a strict hydration protocol was communicated and reinforced during RT, not all patients may have been able to realize our requests accordingly. Furthermore, similar studies at MRI linacs [48] or retrospectively with CBCT [49] questioned the effectiveness of a strict filling protocol for ART in general. A possible alternative is the use of ultrasound-based pre-treatment measurements to minimize deviations in bladder filling [50]. For the entire patient cohort, bladder filling yielded only a weak positive correlation with adaptation time. Undesirable deviations were more dependent on different bladder shape types [51] or on unpredictable pelvic muscle movement.

With respect to constraint violations in rectum there was neither a clear trend towards individual Tx nor towards individual patients. Especially due to the relatively low number of treatment sessions, an endorectal balloon possibly presents an adequate treatment aid to minimize risk for GI complications [52].

Although the employed treatment system possessed a markedly improved CBCT image quality the soft tissue contrast in overlap regions between prostate, urethra, SV, bladder and rectum remains inferior to the one in MRI. Furthermore, the consistency of online segmentation on CBCT1 may have been affected by inter-observer variations. Although this evaluation revealed dose deviations directly after adaptation, no extrapolation for the subsequent dose delivery could be made.

This study used an isotropic 2 mm margin for CTV plus a 2/3 mm margin (posterior/else) for PTV instead of the recommended 3-5/ 4-5mm directly from CTV without extension due to daily adaptations and potential extracapsular tumor extension. Since our study results indicated that in 3 out of 80 treatment sessions (4%) the PTV constraint was not met after adaptation while on the other hand already rectum constraint violations occurred in 10 out of 80 sessions, we refrained from further increasing the posterior CTV-PTV margin. Time-resolved image guidance, obtained from MR cine tracking [53] or ultrasound [54] would be desirable.

In uHF prostate radiation therapy, oART ensured adequate target volume dose coverage, even when the target volume changed in shape or volume during the radiation therapy course while simultaneously fulfilling relevant DVH metrics for bladder and rectum. The adaptation process itself, however, resulted in a subsequent degradation of treatment quality. Since adaptation can compensate for inter-fractional changes, a short overall treatment session combined with intra-fractional motion tracking will ensure robustness for online adaptive prostate SBRT. Future improvements should focus on a higher degree of process automation, a more accurate AI-based image segmentation algorithm and specific training for dedicated personnel. Lastly, clinical benefits should be analyzed in a prospective setting in order to compare oART to conventional IGRT.

## CRediT authorship contribution statement

**Miriam Eckl:** Conceptualization, Data curation, Formal analysis, Investigation, Methodology, Visualization, Writing – original draft. **Nour Alfakhori:** Data curation, Formal analysis, Investigation, Writing – review & editing. **Marvin Willam:** Data curation, Formal analysis, Investigation, Writing – review & editing. **Hans Oppitz:** Data curation, Formal analysis, Writing – review & editing. **Constantin Dreher:** Investigation, Methodology, Resources, Writing – review & editing. **Michael Ehmann:** Conceptualization, Methodology, Writing – review & editing. **Judit Boda-Heggemann:** Investigation, Methodology, Resources, Writing – review & editing. **Frank A. Giordano:** Conceptualization, Methodology, Project administration, Resources, Writing – review & editing. **Jens Fleckenstein:** Conceptualization, Formal analysis, Methodology, Project administration, Resources, Visualization, Writing – original draft.

## Declaration of competing interest

The authors declare that they have no known competing financial interests or personal relationships that could have appeared to influence the work reported in this paper.
