# Supplementary material for: A cone-beam computed tomography based workflow for online adaptive ultra-hypofractionated radiotherapy of prostate cancer
Source: Phys Imaging Radiat Oncol. 2025 Nov 17;36:100869. doi: 10.1016/j.phro.2025.100869 (PMC12681559; doi:10.1016/j.phro.2025.100869)
Supplement: Supplementary Data 1 [file mmc1.docx]

**Table S1**: Spearman's correlation coefficients (r) and statistical significance (p). Bold numbers indicate a statistically significant correlation.

| Variable 1 | Variable 2 | r | p |
| --- | --- | --- | --- |
| Tx | ΔT | 0.50 | 0.39 |
| Tx | Vol(prostate) | **0.98** | **0.01** |
| Tx | Vol(bladder) | -0.30 | 0.62 |
| Tx | Vol(rectum) | 0.30 | 0.62 |
| Vol(CTV_CBCT1_) | DSC(CTV_CBCT1_,CTV_CBCT2_) | **0.30** | **0.01** |
| Vol(CTV_CBCT1_) | ΔV40Gy(CTV)_CBCT1,CBCT2_ | 0.00 | 0.98 |
| ΔT | Vol(bladder) | **0.34** | **0.00** |
| ΔT | ΔV37Gy(bladder) | 0.07 | 0.56 |
| ΔT | ΔV36Gy(rectum) | 0.03 | 0.79 |
